# Spiritually Motivated Self-Forgiveness and Divine Forgiveness, and Subsequent Health and Well-Being Among Middle-Aged Female Nurses: An Outcome-Wide Longitudinal Approach

**DOI:** 10.3389/fpsyg.2020.01337

**Published:** 2020-07-09

**Authors:** Katelyn N. G. Long, Ying Chen, Matthew Potts, Jeffrey Hanson, Tyler J. VanderWeele

**Affiliations:** ^1^Human Flourishing Program, Institute for Quantitative Social Science, Harvard University, Cambridge, MA, United States; ^2^Department of Epidemiology, Harvard T.H. Chan School of Public Health, Boston, MA, United States; ^3^Harvard Divinity School, Cambridge, MA, United States; ^4^Department of Biostatistics, Harvard T.H. Chan School of Public Health, Boston, MA, United States

**Keywords:** forgiveness, religiously or spiritually motivated forgiveness, self-forgiveness, divine forgiveness, health, well-being, outcome-wide epidemiology, mid-life

## Abstract

**Background:** Interest in the relationship between forgiveness and health is steadily growing across disciplines within the research community. While there are multiple forms of forgiveness, past research has focused principally on studying forgiveness of others, whereas longitudinal evidence on the associations between other forms of forgiveness and health remains scarce.

**Methods:** Using longitudinal data from the Nurses’ Health Study II (from the 2008 Trauma Exposure and Post-traumatic Stress Supplementary Survey to 2015 questionnaire wave), this study employed an outcome-wide analytic approach to prospectively examine the association between two forms of religiously or spiritually motivated forgiveness, namely, self-forgiveness and divine forgiveness, and a wide array of subsequent psychosocial well-being, mental health, health behavior, and physical health outcomes among middle-aged female nurses (*N* = 54,703 for self-forgiveness; *N* = 51,661 for divine forgiveness). All models controlled for sociodemographic factors, prior religious service attendance, and prior values of all outcome variables wherever data were available. Bonferroni correction was used to account for multiple testing.

**Results:** Self-forgiveness was strongly associated with greater psychosocial well-being (e.g., for top vs. bottom level of self-forgiveness, β = 0.23, 95% CI: 0.20, 0.25 for positive affect) and lower psychological distress (e.g., β = −0.21, 95% CI: −0.23, −0.18 for depressive symptoms). To a lesser extent, divine forgiveness was also associated with higher levels of psychological well-being and lower psychological distress. For both forgiveness types, there was little evidence of association with physical health or health behavior outcomes, though possible marginal evidence for an association of self-forgiveness with increased mortality.

**Discussion:** This study provides novel evidence that religiously or spiritually motivated self-forgiveness and divine forgiveness are both positively related to several indicators of psychosocial well-being and inversely associated with psychological distress outcomes, whereas the associations with physical health and health behaviors are less clear. Further longitudinal investigation of the dynamics between these types of forgiveness and health and well-being is warranted.

## Introduction

Forgiveness is a concept with a range of meanings and implications across time and place. Varied understandings of forgiveness emerged thousands of years ago from the world’s major religions, including Buddhism, Hinduism, Judaism, Christianity, and Islam (Rye et al., 2000; Tucker et al., 2015). Philosophers past and present have grappled with the definition, conditions, limits, and morality of forgiveness, with increased attention following the Second World War (Voiss, 2015). In the latter half of the twentieth century, psychologists likewise paid growing attention to the concept of forgiveness, particularly for those who had experienced significant personal trauma. These interests, in turn, led to a rising number of empirical studies of forgiveness that gained traction in the late 1980s and early 1990s (Voiss, 2015). To date, interest in the relationship between forgiveness, well-being, and health continues to grow across disciplinary boundaries, including psychology, medicine, and even public health (Toussaint et al., 2015; VanderWeele, 2018).

Among the rising numbers of forgiveness studies, the vast majority explore the impact of forgiving *others* on health and well-being (Toussaint et al., 2015). Yet, other types of forgiveness, such as self-forgiveness and divine forgiveness, warrant investigation both conceptually and empirically. Conceptually, both divine and self-forgiveness are experienced by offenders, requiring a twofold recognition of the self as (1) a moral agent who has failed and (2) a moral agent as valuable, with capacity to change (Worthington and Wade, 2020b); the difference, of course, is the source: God/higher power and self. Some philosophers have called forgiveness a “species of love,” building on Thomas Aquinas’s conception of love as desiring the good and desiring union (Stump, 2006). In this view, divine forgiveness might be understood as that which restores a person and brings him or her into renewed relationship with God/higher power, while self-forgiveness may promote the desire for a good or flourishing life and internally unified relationship with oneself or internal peace (Stump, 2006). Philosopher and theologian Søren Kierkegaard wrote extensively about the dialectic between despair, self-forgiveness, and divine forgiveness, ultimately concluding that genuine reception of and faith in divine forgiveness inescapably requires a person to forgive themselves (Kierkegaard, 1983; Podmore, 2009; Hanson, 2017). Other philosophers and historians note important distinctions between forgiveness and reconciliation (Jackson, 2009; Potts, 2019) and highlight the way that social, religious, and philosophical movements in modern Western history influence notions of self- and divine forgiveness. For example, the Reformation in the sixteenth century and the Enlightenment in the late seventeenth and eighteenth centuries both contributed to Western society’s move away from an emphasis on religious institutional structures and sacramental rites toward an emphasis on the individual and an inward experience of religion (Konstan, 2012). By extension, these historical movements influence concepts like self-forgiveness and divine forgiveness by emphasizing the affective dimension of forgiveness (“I feel forgiven by God/myself”) more so than behavior change predicated on ritualized forms of forgiveness (“because I confessed, I am forgiven and will now do X”).

Mirroring philosophical and theological reflection, empirical work has also explored the relationship between self- and divine forgiveness. To date, studies have found that one’s sense of being forgiven by a higher power is related to increased self-forgiveness (Hall and Fincham, 2008; McConnell and Dixon, 2012), that positive views of the sacred are associated with greater tendency to forgive the self (Davis et al., 2013), and that divine forgiveness may moderate the relationship between self-forgiveness and psychological distress (Fincham and May, 2019). Longitudinal studies have found divine forgiveness to be positively associated with both self-forgiveness and the forgiveness of others in a monotonic pattern; in other words, as divine forgiveness increases, so do the other two types (Chen et al., 2018).

Empirical study, although mostly cross-sectional, has also started to explore the nature and impact of self- and divine forgiveness on human health and well-being. A recent summary of empirical literature suggests that self-forgiveness can be defined as “a process acknowledging and working through one’s responsibility for one’s perceived transgression, but then releasing self-condemnation with its associated emotional, cognitive, and behavioral consequences” (Woodyatt and Wenzel, 2020). Within this view, self-forgiveness is composed of two dimensions: a cognitive component of taking responsibility and working through what has occurred, and an affective component of reducing feelings associated with self-condemnation (Griffin et al., 2018). While measured differently among different studies, a recent review of self-forgiveness literature reported a positive association between self-forgiveness and some mental health outcomes (Massengale et al., 2017), while another meta-analysis reported positive correlations between self-forgiveness with some physical health and psychological well-being outcomes (Davis et al., 2015). Divine forgiveness remains the least common form of forgiveness studied in forgiveness research and is often assessed by a single item (Fincham and May, 2019). The work that has been done has found generally positive relationships between divine forgiveness and psychosocial well-being (Exline, 2020), with less clear relationships to physical health and health behavior (Krause and Ironson, 2017; Chen et al., 2018). While the mechanisms between forgiveness and positive outcomes are generally thought to be beneficial emotion regulation (McCullough et al., 2007; Witvliet and McCullough, 2007), different forms of forgiveness, like self- or divine forgiveness, may influence health and well-being through different mechanisms. One strong commonality between studies on self-forgiveness and divine forgiveness is the call for research that uses longitudinal data to help clarify the relationship between these particular forms of forgiveness and subsequent health and well-being (Exline, 2020; Worthington and Wade, 2020a).

To further investigate understudied forms of forgiveness, this study used an outcome-wide analytic approach (VanderWeele, 2017; VanderWeele et al., 2020) to prospectively examine the association between religiously or spiritually motivated self-forgiveness and divine forgiveness and a wide array of subsequent psychosocial well-being, mental health, health behaviors, and physical health outcomes in a large cohort of middle-aged female nurses in the U.S., controlling for prior values of the outcome variables wherever data were available.

## Materials and Methods

### Study Population

This study used longitudinal data from the Nurses’ Health Study II (NHSII) (Colditz et al., 1997). The NHSII began in 1989 with 116,429 female nurses between the ages of 25 and 42 years, living in 14 US states (Bao et al., 2016). Over the past 30 years, participants in the NHSII completed surveys, either by mail or online, every 2 years, with a response rate over 90% at each follow-up cycle. The NHSII questionnaires cover a wide range of items including exposures in early life, physical activity, health problems, alcohol consumption, body weight profile, diet, mental health, and a range of social, economic, and well-being outcomes (Bao et al., 2016). In 2008, a supplemental survey on Exposure and Post-Traumatic Stress was distributed to a subset of NHSII participants, which included questions about spiritually or religiously motivated forgiveness; thus, this year was considered as the baseline for the present study. Data on the outcome variables were taken from the most recent NHSII questionnaire waves, primarily the 2015 wave; if the outcome was not measured at the 2015 wave, we used data from the 2013 or 2011 wave. All covariates were measured at the 2008 wave or prior waves. We excluded those who reported not believing in God or a higher power from all analyses on divine forgiveness. This yielded an analytic sample of 54,703 participants for analyses on self-forgiveness, and 51,661 participants for analyses on divine forgiveness. Details regarding the sample derivation process were reported in Supplementary Text. This study was approved by the Institutional Review Board at Brigham and Women’s Hospital.

### Measures

#### Forgiveness

Within the 2008 Supplementary Survey on Exposure and Post-Traumatic Stress, the following questions about trait forgiveness (one’s general propensity), derived from the Brief Multidimensional Measure of Religiousness/Spirituality (Idler et al., 2003), were asked: “Because of my spiritual or religious beliefs: (1) I have forgiven myself for the things that I have done wrong, and (2) I know that God or a higher power forgives me.” Each question was answered on a four-point scale (always or almost always, often, seldom, never) with the exception of Divine Forgiveness, which also included the option “Do not believe in God or a higher power.” Those who selected this option were excluded from our analysis on Divine Forgiveness. For the purposes of analysis, we used self-forgiveness and divine forgiveness as two separate exposures, collapsing the bottom two levels of responses due to data sparsity (see Supplementary Table S1 for further details): never/seldom, often, almost always/always. As a sensitivity analysis, we also considered the responses to both types of forgiveness as continuous scores.

#### Outcomes

Using data from the 2011, 2013, or 2015 waves, 19 outcomes were assessed in four categories: (1) psychological well-being (positive affect and social integration); (2) psychological distress (depression diagnosis, depressive symptoms, anxiety symptoms, anxiety diagnosis, hopelessness, and loneliness); (3) health behaviors (heavy drinking, current cigarette smoking, frequent physical activity, preventive healthcare use, dietary quality); and (4) physical health (all-cause mortality, type 2 diabetes, stroke, heart diseases, cancer, overweight/obesity, number of physical health problems; sum of the above five physical illness conditions). Further information for how each of these variables was assessed is available in Supplementary Text.

#### Covariates

Data on all covariates were taken from the 2008 supplementary survey or prior waves. Specifically, a wide range of sociodemographic covariates were controlled for, including age (in years), race (non-Hispanic white, others), marital status (married/in domestic relationship, unmarried), geographic region (Northeast, South, West, Midwest), subjective social standing in US and in community (both rated on a scale ranging from 1 to 10) (Giatti et al., 2012), census tract college education rate (continuous), pre-tax household income (<$50,000, $50,000–$74,999, $75,000–$99,999, ≥$100,000), census tract median income (<$50,000, $50,000–$74,999, $75,000–$99,999, ≥$100,000), night shift work over past 2 years (none, 1–9 months, 10–19 months, 20+ months), employment status (currently employed, non-employed), childhood abuse (summary score 0 to 5), religious service attendance (never/almost never, <1/week, ≥1/week), number of close friends (none, 1–2, 3–5, 6–9, 10 or more), menopausal status (premenopausal or uncertain, postmenopausal), and post-menopausal hormone use (yes, no). In addition, we controlled for prior values of all outcome variables wherever data were available to reduce the possibility of reverse causation (VanderWeele et al., 2016). These include positive affect (continuous score 1–4), depression diagnosis (yes, no), prior depressive symptoms (continuous score ranging from 0 to 30), phobic anxiety (continuous score: 0–16), hopelessness (continuous score: 0–3), alcohol intake (0 g/day, 0.1–9.9 g/day, 10.0–29.9 g/day, ≥30 g/day), smoking status (never smoker, former smoker, current smoker 1–14, 15–24, ≥25 cigarettes/day), physical activity (metabolic equivalents task hours/week: <3, 3–8.9, 9–17.9, 18–26.9, ≥27) (Hu et al., 1999), preventive healthcare use (yes, no), AHEI dietary score (in tertiles) (Chiuve et al., 2012), history of type 2 diabetes (yes, no), stroke (yes, no), heart disease (yes, no), or cancer (yes, no), and body mass index (<25, 25–29.9, ≥30 kg/m^2^).

### Statistical Analysis

All statistical analyses were performed using SAS, version 9.4 (SAS Institute, Inc., Cary, NC, United States). *P*-values were calculated based on two-sided tests. Chi-square tests and analysis of variance tests were used to examine participant characteristics across levels of forgiveness at baseline.

We used an outcome-wide analytic approach (VanderWeele, 2017; VanderWeele et al., 2020) to examine forgiveness in relation to a wide range of health and well-being outcomes simultaneously. This approach fits similar regression models for the relationship between one exposure and multiple outcomes while controlling for similar covariates in each regression. This helps provide a broad picture of the dynamics across a range of outcomes, facilitates the comparison of effect sizes across outcomes within a same sample, reduces “researcher degrees of freedoms” (Simmons et al., 2011) in choosing regression results, facilitates publication of null results, and may help better inform public health recommendations. Further description of this approach was provided elsewhere (VanderWeele, 2017; VanderWeele et al., 2020). Following this approach, we ran a separate regression model for each forgiveness type and outcome. Depending on the nature of the outcome variable, we ran one of three different models: (1) logistic regressions for binary outcomes with a prevalence <10% to estimate odds ratios; for rare outcomes, odds ratios would approximate risk ratios; (2) Poisson regression models for binary outcomes with a prevalence ≥ 10% to estimate risk ratios (Zou, 2004); and (3) linear regression models for continuous outcomes to estimate beta. With continuous variables, we standardized outcomes (mean = 0, standard deviation = 1) to allow effect sizes to be interpreted in terms of standard deviation change in the outcome variable, which also facilitated comparison of effect estimates across outcomes. All models were fully adjusted for all covariates. Bonferroni correction was used to adjust for multiple testing.

#### Multiple Imputation

Multiple imputation with a chained equations procedure (five imputed datasets were generated) was used to impute for missing data on all variables. Multiple imputation often produces less biased estimates as compared to other methods of handling missing data (Moons et al., 2006; Sterne et al., 2009; Groenwold et al., 2012).

#### Sensitivity Analyses

First, to evaluate potential unmeasured confounding, we performed sensitivity analysis using *E*-values (VanderWeele and Ding, 2017; Mathur et al., 2018), which assesses the minimum strength that an unmeasured confounder would have to have on the risk ratio scale with *both* the exposure (self- or divine forgiveness) and the outcome to explain away the association. Next, we reanalyzed the primary sets of models using complete-case analysis. Third, to further reduce concerns of reverse causation, we reanalyzed both forms of forgiveness, restricting to participants who were *free*, at baseline, of the four major physical health problems in our study (type 2 diabetes, stroke, heart disease, and cancer). While controlling for prior illness was the method in our main analysis, our supplementary analysis removing people with all four major illnesses provided more conservative estimates as people who are already sick might be more likely to forgive themselves or accept forgiveness from God or a higher power. To examine forgiveness in relation to the *incidence*, or first-time occurrence, of each type of physical illness, we reanalyzed the models of physical illness outcomes, excluding participants with each condition (one by one) at baseline. In other words, we only included people who had *not* been diagnosed with diabetes at baseline (although they may have had one of the other four conditions) to examine the incidence of diabetes in the later waves of the study. Finally, we also considered the responses to both forms of forgiveness as continuous scores, to compare with our main analysis, which assessed forgiveness as categorical variables.

## Results

### Descriptive Analyses

Participant characteristics in the full sample are shown in Supplementary Table S1. At baseline, the average age of respondents was 53.37 years old (*SD* = 4.65). The majority of the respondents were non-Hispanic White (95.75%), married (81.41%), currently employed (88.78%), and had relatively high SES. The distribution of participant characteristics by levels of self-forgiveness is reported in Table 1, and that by levels of divine forgiveness is shown in Supplementary Table S2.

**TABLE 1:** Participant characteristics (age-adjusted) by self-forgiveness at study baseline (The Nurses’ Health Study II 2008 Supplementary Survey, *N* = 53,226).

	Self-forgiveness		
Participant characteristics	Never/seldom (*n* = 7424)	Often (*n* = 23,621)	Always/almost always (*n* = 22,181)
Age, in years (range: 43–64)^a^	53.22 (4.62)	53.32 (4.66)	53.41 (4.65)
Non-hispanic white, %	96.40	95.95	95.30
Marital status, %	77.61	80.99	83.28
**Geographic region, %**			
- Northeast	38.08	33.19	28.36
- Midwest	29.59	33.21	34.29
- South	15.20	18.11	21.03
- West	17.13	15.48	16.32
Subjective SES in US (range: 1–10)	6.81 (1.42)	7.05 (1.28)	7.26 (1.30)
Subjective SES in community (range: 1–10)	6.46 (1.71)	6.84 (1.54)	7.17 (1.53)
Census-tract college education rate (range: 0–0.88)	0.33 (0.16)	0.32 (0.16)	0.31 (0.16)
**Household income, %**			
- <$50,000	17.27	15.76	15.40
- $50,000–$74,999	27.24	27.71	27.30
- $75,000–$99,999	20.84	21.57	21.52
- ≥$100,000	34.66	34.96	35.78
**Census tract median income, %**			
- <$50,000	24.35	25.30	27.05
- $50,000–$74,999	47.59	49.64	48.95
- $75,000–$99,999	20.48	18.97	18.35
- ≥$100,000	7.58	6.09	5.66
**Night shift work over past 2 years, %**			
- None	90.95	91.82	92.66
- 1–9 months	4.08	3.56	3.03
- 10–19 months	1.30	1.38	1.21
- 20+ months	3.67	3.23	3.10
Currently employed, %	89.11	89.57	87.84
Childhood abuse victimization (range: 0–5)	2.04 (1.56)	1.78 (1.48)	1.65 (1.48)
**Religious service attendance, %**			
- Never/almost never	38.52	21.70	17.91
- <Once/week	41.60	39.71	30.65
- ≥Once/week	19.88	38.60	51.44
Number of close friends (range: 0–5)	1.52 (0.72)	1.72 (0.64)	1.81 (0.65)
Depressive symptoms (range: 0–30)	9.42 (6.03)	6.33 (4.74)	4.63 (4.24)
Depression diagnosis, %	23.84	15.16	12.31
Anxiety symptoms (range: 0–15)	3.16 (2.60)	2.55 (2.21)	2.07 (2.02)
Hopelessness (range: 0–3)	1.30 (0.93)	0.90 (0.88)	0.67 (0.90)
Positive affect (range: 0–3)	1.68 (0.81)	2.05 (0.72)	2.29 (0.73)
Preventive healthcare use, %	81.75	85.47	86.34
**Alcohol intake, %**			
- 0 g/day	31.59	31.18	37.35
- 0.1–9.9 g/day	44.25	46.48	42.51
- 10.0–29.9 g/day	18.64	18.40	16.62
- 30+ g/day	5.52	3.94	3.53
**Cigarette smoking, %**			
- Never smoker	59.86	65.57	68.70
- Former smoker	31.76	28.35	26.17
- Current smoker 1–14/day	4.34	3.42	2.91
- Current smoker 15–24/day	2.88	1.98	1.71
- Current smoker ≥ 25/day	1.16	0.67	0.51
**Physical activity (METS^b^), %**			
- <3	19.32	15.56	15.36
- 3–8.9	20.01	18.97	18.75
- 9–17.9	19.10	21.07	20.78
- 18–26.9	12.77	13.87	14.63
- ≥27	28.79	30.52	30.49
**Dietary quality (AHEI score^c^), %**			
- Bottom tertile, %	36.24	32.57	31.10
- Middle tertile, %	32.99	34.02	32.89
- Top tertile, %	30.77	33.41	36.01
**BMI categories (kg/m^2^), %**			
- <20	6.01	5.46	5.20
- 20–24.9	35.77	38.10	38.65
- 25–29.9	28.58	29.53	29.95
- 30–34.9	15.40	15.22	15.12
- 35+	14.24	11.69	11.09
Diabetes, %	5.12	4.58	4.57
CHD, %	1.46	1.07	1.13
Stroke, %	1.69	1.27	1.29
Cancer, %	6.62	6.61	6.48
Postmenopausal status, %	60.74	60.23	60.81
Replacement hormone use, %	14.04	14.34	14.64

*Values are means (SD) for continuous variables and percentages for categorical variables, and are standardized to the age distribution of the study population. Values of polytomous variables may not sum to 100% due to rounding. ^*a*^Value is not age adjusted. ^*b*^Metabolic equivalents score (METS) was used to measure physical activity. ^*c*^Alternate Healthy Eating Index (AHEI) was used to measure dietary quality.*

### Self-Forgiveness and Subsequent Health and Well-Being

Participants who reported the highest level of self-forgiveness (vs. the lowest level) had higher psychological well-being in the domains of positive affect (β = 0.23; 95% CI: 0.20, 0.25) and social integration (β = 0.11; 95% CI: 0.09, 0.13) (Table 2). Further, the highest vs. the lowest level of self-forgiveness was associated with nearly all outcomes of psychological distress such as fewer depressive symptoms (β = −0.21; 95% CI: −0.23, −0.18) and lower levels of hopelessness (β = -0.18; 95% CI: −0.21, −0.15). However, there was little evidence of association with health behaviors and physical health outcomes, with the exception of all-cause mortality where the highest vs. the lowest level of self-forgiveness was associated with an increased risk of mortality (RR = 1.33; 95% CI: 1.04, 1.71) (Table 2), though this association did not pass a *p* = 0.05 threshold after Bonferroni correction. The association was also attenuated in our sensitivity analysis that was restricted to participants free of major physical health problems at baseline (Supplementary Table S3). The sensitivity analysis using complete-case analyses yielded similar results (Supplementary Table S4). The sensitivity analysis examining incidence of physical health outcomes also suggested no evidence of association between self-forgiveness and subsequent physical health (Supplementary Table S5). Further, the sensitivity analysis considering self-forgiveness as a continuous variable also yielded similar results to our primary analysis (Supplementary Table S6).

**TABLE 2:** Self-forgiveness and subsequent health and well-being in mid-life (The Nurses’ Health Study II 2008 supplementary survey to 2011, 2013, or 2015 questionnaire wave, *N* = 54,703^a^).

	Self-forgiveness^b^							

	Often vs. Never/seldom				Always/almost always vs. Never/seldom			
Health and well-being outcomes	RR^c^	β^d^	95% CI	*P*-value threshold	RR^c^	β^d^	95% CI	*P*-value threshold
**Psychosocial well-being**								
Positive affect		0.12	0.10, 0.15	<0.0026^e^		0.23	0.20, 0.25	<0.0026^e^
Social integration		0.07	0.05, 0.09	<0.0026^e^		0.11	0.09, 0.13	<0.0026^e^
**Psychological distress**								
Depression diagnosis	0.98		0.91, 1.06		0.93		0.85, 1.01	
Depressive symptoms		–0.13	−0.15, −0.10	<0.0026^e^		–0.21	−0.23, −0.18	<0.0026^e^
Anxiety symptoms		–0.01	−0.04, 0.02			–0.15	−0.18, −0.12	<0.0026^e^
Hopelessness		–0.12	−0.15, −0.10	<0.0026^e^		–0.18	−0.21, −0.15	<0.0026^e^
Loneliness		–0.10	−0.12, −0.07	<0.0026^e^		–0.12	−0.15, −0.10	<0.0026^e^
**Health behaviors**								
Heavy drinking	0.97		0.83, 1.13		0.97		0.82, 1.15	
Current cigarette smoking	0.94		0.79, 1.11		0.95		0.79, 1.14	
Frequent physical activity	1.00		0.97, 1.04		1.00		0.97, 1.04	
Preventive healthcare use	1.00		0.97, 1.03		0.99		0.96, 1.02	
Dietary quality		0.00	−0.02, 0.02			0.02	−0.01, 0.04	
**Physical health**								
All-cause mortality	1.12		0.89, 1.42		1.33		1.04, 1.71	<0.05
No. of physical health problems		0.00	−0.02, 0.02			0.01	−0.01, 0.03	
Diabetes	0.88		0.76, 1.01		0.97		0.83, 1.13	
Stroke	1.08		0.80, 1.46		1.01		0.73, 1.39	
Heart disease	0.89		0.61, 1.30		1.41		0.95, 2.07	
Cancer	0.95		0.88, 1.03		0.97		0.89, 1.05	
Overweight/obesity	1.02		0.98, 1.05		1.02		0.98, 1.06	

*RR, risk ratio; CI, confidence interval. ^*a*^The full analytic sample was restricted to those who responded to the Nurses’ Health Study II 2008 supplementary survey in which the exposure variable forgiveness was assessed. Multiple imputation was performed to impute missing data on all variables. ^*b*^A set of generalized estimating equations were used to regress each outcome on forgiveness separately. All models controlled for participants’ age, race, marital status, geographic region, childhood abuse, socioeconomic status (subjective SES, household income, census tract college education rate, and census tract median income), employment status, night shift work schedule, religious service attendance, number of close friends, and prior health status or health behaviors (prior depressive symptoms, depression diagnosis, anxiety symptoms, hopelessness, positive affect, dietary quality, body mass index, smoking, alcohol intake, physical activity, preventive healthcare use, postmenopausal status, menopausal hormone therapy use, history of diabetes, heart diseases, stroke, and cancer). ^*c*^The effect estimates for the outcomes of heavy drinking, current smoking, mortality, diabetes, heart diseases, stroke, and cancer were odds ratio. These outcomes were rare (prevalence < 10%), so the odds ratio would approximate RR. Effect estimates for other dichotomized outcomes were RR. ^*d*^All continuous outcomes were standardized (mean = 0, standard deviation = 1), and β was the standardized effect size. ^*e*^p < 0.05 after Bonferroni correction (the p-value cutoff for Bonferroni correction is p = 0.05/19 outcomes = 0.0026).*

### Divine Forgiveness and Subsequent Health and Well-Being

To a lesser extent, divine forgiveness was also positively associated with psychological well-being (e.g., positive affect, β = 0.19; 95% CI: 0.15, 0.22) and was inversely associated with psychological distress (e.g., depressive symptoms, β = −0.15; 95% CI: −0.18, −0.12; hopelessness, β = −0.16; 95% CI: −0.20, −0.12) (Table 3). Among health behaviors and physical health outcomes, there were few associations, with the exception of some evidence that divine forgiveness was related to higher risk of overweight obesity (RR = 1.06; 95% CI: 1.00, 1.11) and more physical health problems (β = 0.03; 95% CI: 0.00, 0.05). However, these associations did not reach a *p*-value threshold smaller than 0.05 after Bonferroni correction (Table 3). The complete-case analyses (Supplementary Table S7) and the sensitivity analysis restricting to participants free of major physical health problems at baseline both yielded similar results (Supplementary Table S8). The sensitivity analysis that examined incidence of physical health outcomes also suggested little evidence of association with divine forgiveness with the exception of a stronger association with incidence of overweight/obesity as compared to the main model (RR = 1.40; 95% CI: 1.17, 1.68) (Supplementary Table S5). It is also interesting to note that although most confidence intervals overlapped the null, point estimates for incident diabetes, heart disease, and cancer were in the opposite direction for self- vs. divine forgiveness. The sensitivity analysis assessing divine forgiveness as a continuous variable also yielded similar results to the primary analysis (Supplementary Table S9).

**TABLE 3:** Divine forgiveness and subsequent health and well-being in mid-life (The Nurses’ Health Study II 2008 supplementary survey to 2011, 2013, or 2015 questionnaire wave, *N* = 51,661^a^).

	Divine forgiveness^b^							

	Often vs. Never/seldom				Always/almost always vs. Never/seldom			
Health and well-being outcomes	RR^c^	β^d^	95% CI	*P*-value threshold	RR^c^	β^d^	95% CI	*P*-value threshold
**Psychosocial well-being**								
Positive affect		0.09	0.05, 0.13	<0.0026^e^		0.19	0.15, 0.22	<0.0026^e^
Social integration		0.03	0.00, 0.05			0.12	0.09, 0.15	<0.0026^e^
**Psychological distress**								
Depression diagnosis	1.03		0.92, 1.16		1.06		0.94, 1.18	
Depressive symptoms		–0.08	−0.12, −0.04	<0.0026^e^		–0.15	−0.18, −0.12	<0.0026^e^
Anxiety symptoms		0.03	−0.01, 0.08			–0.03	−0.07, 0.01	
Hopelessness		–0.07	−0.12, −0.03	<0.0026^e^		–0.16	−0.20, −0.12	<0.0026^e^
Loneliness		–0.06	−0.10, −0.02	<0.01		–0.11	−0.15, −0.07	<0.0026^e^
**Health behaviors**								
Heavy drinking	0.97		0.79, 1.21		1.06		0.87, 1.30	
Current cigarette smoking	1.20		0.92, 1.56		1.09		0.85, 1.40	
Frequent physical activity	0.99		0.95, 1.04		0.98		0.93, 1.03	
Preventive healthcare use	1.01		0.97, 1.05		1.01		0.97, 1.05	
Dietary quality		–0.03	−0.06, 0.01			–0.03	−0.06, 0.00	
**Physical health**								
All-cause mortality	0.88		0.62, 1.24		1.05		0.76, 1.46	
No. of physical health problems		0.02	−0.01, 0.04			0.03	0.00, 0.05	<0.05
Diabetes	0.89		0.71, 1.10		0.89		0.73, 1.09	
Stroke	0.87		0.54, 1.40		1.05		0.68, 1.63	
Heart disease	0.71		0.42, 1.22		0.73		0.44, 1.20	
Cancer	0.97		0.87, 1.09		0.98		0.87, 1.09	
Overweight/obesity	1.05		0.99, 1.11		1.06		1.00, 1.11	<0.05

*RR, risk ratio; CI, confidence interval. ^*a*^The full analytic sample was restricted to those who responded to the Nurses’ Health Study II 2008 supplementary survey in which the exposure variable forgiveness was assessed. Participants who reported not believing in God or a higher power were removed from the analyses. Multiple imputation was performed to impute missing data on all variables. ^*b*^A set of generalized estimating equations were used to regress each outcome on forgiveness separately. All models controlled for participants’ age, race, marital status, geographic region, childhood abuse, socioeconomic status (subjective SES, household income, census tract college education rate, and census tract median income), employment status, night shift work schedule, religious service attendance, number of close friends, prior health status, or health behaviors (prior depressive symptoms, depression diagnosis, anxiety symptoms, hopelessness, positive affect, dietary quality, body mass index, smoking, alcohol intake, physical activity, preventive healthcare use, postmenopausal status, menopausal hormone therapy use, history of diabetes, heart diseases, stroke, and cancer). ^*c*^The effect estimates for the outcomes of heavy drinking, current smoking, mortality, diabetes, heart diseases, stroke and cancer were odds ratio. These outcomes were rare (prevalence < 10%), so the odds ratio would approximate RR. Effect estimates for other dichotomized outcomes were RR. ^*d*^All continuous outcomes were standardized (mean = 0, standard deviation = 1), and β was the standardized effect size. ^*e*^p < 0.05 after Bonferroni correction (the p-value cutoff for Bonferroni correction is p = 0.05/19 outcomes = 0.0026).*

### Sensitivity Analyses on Unmeasured Confounding

Although we adjusted for a range of potential confounding variables, this study used observational data and thus there may still have been uncontrolled confounding, for example, aspects of personality, or the experience of a recent major offense. Controlling for baseline outcomes partially helps to mitigate bias from such uncontrolled confounding. However, we also report *E*-values to assess sensitivity or robustness of results to potential unmeasured confounding. *E*-values suggested that several of the associations we observed were at least somewhat robust to unmeasured confounding (Table 4). For example, only an unmeasured confounder associated with both self-forgiveness and higher levels of positive affect by risk ratios of 1.77 each, above and beyond the large array of covariates already adjusted for, could suffice to explain away the observed association, but weaker confounding could not. To shift the CI to include the null, an unmeasured confounder associated with both self-forgiveness and higher levels of positive affect by risk ratios of 1.70 each could suffice, but weaker confounding could not. However, for other associations, such as between divine forgiveness and the number of health problems, relatively modest levels of confounding could explain away the association (*E*-value = 1.20 for estimate and 1.08 for CI).

**TABLE 4:** Robustness to unmeasured confounding (*E*-values^a^) for assessing the associations between forgiveness and health and well-being (The Nurses’ Health Study II 2008 Supplementary Survey to 2011, 2013, or 2015 Questionnaire Wave).

Health and well-being outcomes	Self-forgiveness (always/almost always vs. never/seldom)		Divine forgiveness (always/almost always vs. never/seldom)	
	For effect estimate^b^	For CI limit^c^	For effect estimate^b^	For CI limit^c^
Positive affect	1.77	1.70	1.66	1.56
Social integration	1.45	1.39	1.47	1.40
Depression diagnosis	1.36	1.00	1.31	1.00
Depressive symptoms	1.72	1.65	1.56	1.46
Anxiety symptoms	1.56	1.48	1.20	1.00
Hopelessness	1.64	1.56	1.58	1.48
Loneliness	1.47	1.39	1.45	1.33
Heavy drinking	1.25	1.00	1.31	1.00
Current cigarette smoking	1.21	1.00	1.40	1.00
Frequent physical activity	1.00	1.00	1.16	1.00
Preventive healthcare use	1.11	1.00	1.11	1.00
Dietary quality	1.16	1.00	1.20	1.00
All-cause mortality	1.99	1.24	1.28	1.00
No. of physical health problems	1.11	1.00	1.20	1.08
Diabetes	1.25	1.00	1.50	1.00
Stroke	1.32	1.00	1.28	1.00
Heart disease	2.17	1.00	2.08	1.00
Cancer	1.21	1.00	1.16	1.00
Overweight/obesity	1.16	1.00	1.31	1.00

*CI, confidence interval. ^*a*^See VanderWeele and Ding (2017) for the formula and Mathur et al. (2018) for the website and R package for calculating E-values. ^*b*^The E-values for effect estimates are the minimum strength of association on the risk ratio scale that an unmeasured confounder would need to have with both the exposure and the outcome, above and beyond the measured covariates, to fully explain away the observed association of forgiveness (always/almost always vs. never/seldom) with various outcomes. ^*c*^The E-values for the limit of the 95% confidence interval closest to the null denote the minimum strength of association on the risk ratio scale that an unmeasured confounder would need to have with both the exposure and the outcome, above and beyond the measured covariates, to shift the confidence interval to include the null value.*

## Discussion

### Psychosocial Well-Being and Psychological Distress

In this study, religiously or spiritually motivated self-forgiveness and divine forgiveness had positive associations with psychosocial well-being, findings that align with a number of prior studies. For example, a 2015 meta-analysis of self-forgiveness examined 65 studies (largely cross-sectional) and reported positive correlation between self-forgiveness and psychosocial well-being, with self-forgiveness accounting for approximately 20% of the variance in psychological well-being aggregating across measures of depression, anxiety, life satisfaction, and general mental health (Davis et al., 2015). Building off of this study, a follow-up qualitative analysis of self-forgiveness literature, similarly, found that of 60 studies measuring trait self-forgiveness, 59 were robustly linked to mental health, while only one study among adult women with significant trauma reported a null relationship. In our study, the one-item measure of religiously or spiritually motivated self-forgiveness was also associated with improved psychosocial well-being and reduced psychological distress; however, the use of longitudinal data allows for stronger interpretation of results than previous cross-sectional work. Of course, single-item measures are limited in their depth and interpretive potential. However, recent studies employing more nuanced measures of self-forgiveness (e.g., differentiating self-forgiveness from self-exoneration) suggest that both genuine self-forgiveness and self-exoneration were associated with increased well-being (Cornish et al., 2018). One of the few prospective longitudinal studies examining the impact of self-forgiveness on both offender and victim found that genuine self-forgiveness (involving effort to work through one’s offense, responsibility-taking, and self-acceptance while acknowledging failure) was associated with positive restorative outcomes for both parties (Woodyatt and Wenzel, 2013). While these studies examine more robust measures of self-forgiveness, the findings align with our study as those who reported the highest levels of religiously or spiritually motivated self-forgiveness had increased levels of social integration and reduced levels of loneliness.

In our study, those who reported the highest levels of divine forgiveness had better psychosocial well-being than those who reported the lowest levels of divine forgiveness, broadly aligning with the small amount of existing evidence on divine forgiveness. For example, a number of cross-sectional studies examining divine forgiveness have found generally positive relationships with well-being and psychological adjustment (Exline, 2020) such as successful aging among older adults (Lawler-Row, 2010), stronger sense of purpose (Lyons et al., 2011), and lower end-of-life anxiety (Krause, 2015). Others note that while divine forgiveness is not as strongly predictive of better mental health as much as the forgiveness of others (Toussaint et al., 2001; Krause and Ellison, 2003), belief in God’s forgiveness is a strong predictor of unconditional forgiveness of others, which is associated with better mental health outcomes (Krause and Ellison, 2003; Uecker et al., 2016). While a recent longitudinal analysis among a sample of older adult Christians in the US did not find association between divine forgiveness and psychological well-being (optimism, self-esteem, and life satisfaction), it did find that divine forgiveness improved psychological well-being more among those who were securely attached to God (Kent et al., 2018). Another cross-sectional study exploring beliefs in human sinfulness, divine forgiveness, and mental health found that belief in human sinfulness was not a significant impediment to good mental health among those who frequently feel God’s forgiveness, but was related to poor mental health among those who feel God’s forgiveness less frequently (Uecker et al., 2016). As our study only used a single measure of divine forgiveness, it was not possible to assess personal religious beliefs about human relationships to the divine, other than excluding those who specifically stated they did not believe in god or a higher power. However, the associations between divine forgiveness and subsequent psychological well-being and reduced psychosocial distress indicate that divine forgiveness may play a positive role in improved psychosocial well-being among those who do believe in a divine being or force.

### Health Behaviors and Physical Health

Our study found few associations between self- or divine forgiveness and physical health. With regard to self-forgiveness, recent meta-analyses of mostly cross-sectional studies have found weak to moderate associations between self-forgiveness and physical health that decrease with age and when more males were included in the sample (Davis et al., 2015). These results might suggest that a mid-aged female-only sample might yield stronger associations between self-forgiveness and physical health, yet in our analysis, there was little evidence of association with physical health or health behavior outcomes with the possible exception of an increased risk of mortality, though this did not pass the *p* < 0.05 threshold after Bonferroni correction for multiple testing. Despite the weakness of this association in our study, others have noted the potentially “dark side” of self-forgiveness (Thompson, 2011; Woodyatt and Wenzel, 2020). For example, studies found that state self-forgiveness (forgiveness for particular events) was associated with reduced efforts to change or reconcile, particularly when change was hard or emotionally uncomfortable (Cornish et al., 2018; Woodyatt and Wenzel, 2020). In other words, forgiving oneself too easily potentially undermines mechanisms that promote behavior change and maintain healthy relationships, in turn potentially perpetuating cycles of chronic destructive behavior (Thompson, 2011; Davis et al., 2015; Griffin, 2016). Theologians likewise reflect on the need for forgiveness to be a reminder of what right union and relationships look like; “a gracious irritant” that avoids complacency and inspires right action (Jones, 1995). Thus, the associations between self-forgiveness and physical health and health behaviors may merit further study.

In our study, divine forgiveness and physical health only had mild (pre-Bonferroni correction) associations for two outcomes: number of physical health problems and overweight/obesity. Interestingly, in our supplementary analysis examining forgiveness and incidence of physical health problems, the association between divine forgiveness and overweight/obesity was more pronounced with those who report always/almost always receiving divine forgiveness 40% more likely to develop overweight/obesity at follow-up in 2015. While there are very few studies that specifically examine the relationship between divine forgiveness and physical health, a recent outcome-wide longitudinal study (Chen et al., 2018) also found a mild association between divine forgiveness and overweight/obesity among young adults, and a cross-sectional study with US adults found forgiveness by God related to less favorable waist/hip ratios and less frequent exercise among those who were less committed to their faith (Krause and Ironson, 2017). Given the paucity of longitudinal studies related to divine forgiveness (relative to the forgiveness of others and even self-forgiveness) and the relatively low number of studies examining the relationship between forgiveness and physical health (Toussaint et al., 2020), our study offers early evidence regarding the relationship between divine forgiveness and physical health, which we hope catalyzes others to explore further.

### Limitations and Strengths

This study was limited by the use of single-item measures of religiously or spiritually motivated self- and divine forgiveness, which only addressed the emotional component of trait forgiveness (Woodyatt and Wenzel, 2020) and were potentially complicated by the qualifying statement, “Because of my spiritual or religious beliefs…”. To help mitigate this challenge, we removed participants who selected “Do not believe in God or a higher power” in our analysis on divine forgiveness. Future longitudinal studies might consider expanding the assessment of forgiveness with validated forgiveness scales that include a self-forgiveness component, including Enright Forgiveness Inventory (Enright, 1996), Heartland Forgiveness Scale (Thompson and Snyder, 2003), State Self-Forgiveness Scale (Wohl et al., 2008), and the more recently developed Differentiated Process Scale of Self-Forgiveness (Woodyatt and Wenzel, 2013) and Dual Process of Self-Forgiveness Scale (Griffin et al., 2018). For divine forgiveness, considerations may include the multi-dimensional scale assessing divine forgiveness for a specific offense (Martin, 2008) or a multi-item scale on the conditionality of God’s forgiveness (Akl and Mullet, 2010).

The study also had a limited follow-up period, which may not have been enough time to assess the relationship between forgiveness and physical health, particularly the incidence of chronic conditions that tend to develop slowly over time and in later life. The study also included a largely homogeneous sample of white female nurses, which, although quite large, greatly limits the applicability of findings to the general US population as well as populations in other global contexts. As with all observational studies, ours was potentially limited by confounding due to unmeasured factors. However, our use of prospective data, rigorous covariate control, and the sensitivity analyses for unmeasured confounding may help to reduce such concerns.

This study has a number of strengths worth noting. The most critical of these is the use of longitudinal data with a large cohort, which helps address a long-standing gap in forgiveness-related research. By adjusting for prior values of covariates and outcomes in our primary analysis, we were able to reduce concerns of reverse causation, which ultimately provide stronger evidence of causality (Danaei et al., 2012; Hernán, 2015; VanderWeele et al., 2016). We examined multiple associations simultaneously, creating scope to report on strong associations as well as weak or null associations (VanderWeele, 2017; VanderWeele et al., 2020), which are often excluded from publication due to bias toward “significant” findings. Our analysis was further strengthened by our supplementary analyses, which found largely similar associations across methodologies. Additionally, we included a number of physical health and health behavior outcomes, a number of which were objectively assessed, reducing the potential of bias due to self-reported data. For example, physical conditions were verified by medical chart review, and psychosocial outcomes are assessed using validated measures. Finally, this study examines two forms of forgiveness that are relatively understudied in the forgiveness research landscape, which allows this study to make a novel contribution both methodologically and conceptually.

### Implications

Findings in this study highlight the potential that religiously or spiritually motivated self-forgiveness and divine forgiveness may have in improving psychosocial well-being and reducing psychological distress. Recent studies highlight the potential of workbook and counseling interventions to improve self-forgiveness and improve other measures of well-being such as lower self-condemnation, psychological distress, and pessimism, increased drinking refusal, and greater compassion (Toussaint et al., 2014; Cornish and Wade, 2015; Griffin et al., 2015; Bell et al., 2017). However, much remains unknown about the impact of explicit self-forgiveness interventions (Griffin et al., 2018), and even less is known about effective interventions to improve divine forgiveness, although it is likely that such interventions would be best placed in the context of religious teachings suitable to a person’s faith tradition, and perhaps integrated with teaching on other forms of forgiveness given their seeming interdependence (Exline, 2020).

While there seems to be mounting evidence for the benefits of self-forgiveness and divine forgiveness on psychosocial well-being, findings from this study indicate an ambiguous relationship between these forms of forgiveness and physical health, indicating, above all, the need for further evidence to better understand the nature of these relationships. While this study makes a strong contribution in its use of longitudinal data and rigorous analysis, longitudinal data from more diverse cohorts, more robust measures of self-and divine forgiveness, and a better understanding of underlying mechanisms are required.

## Conclusion

This study provides novel evidence that religiously or spiritually motivated self-forgiveness and divine forgiveness are both positively related to several indicators of psychosocial well-being and inversely associated with psychological distress outcomes, whereas the associations with physical health and health behaviors are less clear. Further longitudinal investigation of the dynamics between these types of forgiveness and health and well-being is warranted.

## Data Availability Statement

The data analyzed in this study was obtained from the Nurses’ Health Study II. These are not publicly available due to restrictions set by the data holder but may be made available upon request and permission from the Channing Division of Network Medicine at Brigham and Women’s Hospital. Further information including the procedures to obtain and access data from the Nurses’ Health Study is described at https://www.nurseshealthstudy.org/researchers (email: nhsaccess@channing.harvard.edu).

## Ethics Statement

This study was approved by the Institutional Review Board at Brigham and Women’s Hospital, Boston, MA, United States. The patients/participants provided their written informed consent to participate in this study.

## Author Contributions

TV and YC developed the study concept. KL, YC, MP, JH, and TV contributed to the study design and interpretation of results. YC had full access to the data in the study and takes responsibility for the integrity of the data and accuracy of the data analysis. KL drafted the manuscript. YC, MP, JH, and TV provided critical revisions and approved the final submitted version of the manuscript. All authors contributed to the article and approved the submitted version.

## Conflict of Interest

The authors declare that the research was conducted in the absence of any commercial or financial relationships that could be construed as a potential conflict of interest.
