# Supplementary material for: Spiritually Motivated Self-Forgiveness and Divine Forgiveness, and Subsequent Health and Well-Being Among Middle-Aged Female Nurses: An Outcome-Wide Longitudinal Approach
Source: Front Psychol. 2020 Jul 9;11:1337. doi: 10.3389/fpsyg.2020.01337 (PMC7363844; doi:10.3389/fpsyg.2020.01337)
Supplement: Supplementary file 1 [file Data_Sheet_1.pdf]

SUPPLEMENTARY TEXT

Sample Derivation

This study used longitudinal data from the Nurses' Health Study II (NHSII) (Colditz, Manson, & Hankinson, 1997). The NHSII began in 1989 with 116,429 female nurses between the ages of 25-42, living in 14 US states (Bao et al., 2016). Over the past thirty years, participants in the NHSII completed surveys, either by mail or online, every two years, with a response rate over 90% at each follow-up cycle. The NHSII questionnaires cover a wide range of items including exposures in early life, physical activity, health problems, alcohol consumption, body fat profile, diet, mental health, and a range of social, economic, and well-being outcomes (Bao et al., 2016).

The analytic sample of the present study was drawn from participants of the NHSII 2008 Exposure and Post-Traumatic Stress Supplemental Survey (N=54,703) which included questions on spiritually or religiously motivated forgiveness; thus, this year was considered as the baseline for the present study. Data on the outcome variables were taken from the most recent NHSII questionnaire waves, primarily the 2015 wave; if the outcome was not measured at the 2015 wave, we used data from the 2013 or 2011 wave. In the sample for analyses on self-forgiveness, 1477 participants had missing data on self-forgiveness, another 11,479 with missing data on covariates, and the number of participants with missing data or were lost to follow-up on the outcomes variables ranged from 0 to 4,998 individuals, depending on the outcome. Multiple imputation was performed to impute missing data on all variables. This yielded an analytic sample of 54,703 participants for analyses on self-forgiveness. In the sample for analyses on divine-forgiveness, 971 participants had missing data on divine-forgiveness, another 11,587 with missing data on covariates, and the number of participants with missing data or were lost to follow-up on the outcomes variables ranged from 0 to 5,034 individuals, depending on the outcome. Multiple imputation was performed to impute missing data on all variables. We excluded participants who reported not believing in God or a higher power from analyses on divine forgiveness. This yielded an analytic sample of 51,661 participants for the analyses on divine forgiveness.

Further information about the Nurses' Health Study is available on the website (https://www.nurseshealthstudy.org/). This study was approved by the Institutional Review Board at Brigham and Women's Hospital.

Outcome Assessment

Positive affect. To assess positive affect, an item from the CESD-10 scale (Andresen, Malmgren, Carter, & Patrick, 1994) was used: "I was happy." Response ranged from 0 (rarely or none of the time) to 3 (all the time). We treated the response to this question as a continuous score, with a higher score representing greater positive affect.

Social integration. The simplified Berkman-Syme Social Network Index was used to measure social integration (Berkman & Syme, 1979). Three self-reported aspects of social integration included marital status (married, others), number of close friends (none, 1-2, 3-9, 10+), and participation in community, volunteer groups or other group organizations (none, 1-2 hours/week, 3-10 hours/week, and 11+ hours/week). Religious service attendance was also considered in the original index, however, as service attendance is an exposure variable in our study, we did not include it in the measure. Scoring criteria followed prior work (Chang et al., 2017). An overall score was derived (ranging from 0 to 9) by summing scores across the three dimensions.

Depression. Depression diagnosis was self-reported as yes or no. In addition, depressive symptoms over the past week were assessed using the validated CESD-10 (Andresen et al., 1994) (e.g., "I was bothered by things that usually don't bother me"). Responses ranged from 0 (rarely or none of the time) to 3 (all of the time). When necessary, reverse coding was used such that higher scored indicated more depressive symptoms, and an overall score was calculated using the sum across all items ($\alpha = 0.84$, range=0 to 30).

Anxiety symptoms. The 7-item Generalized Anxiety Disorder Scale was used to assess anxiety symptoms over the past 4 weeks (Spitzer, Kroenke, Williams, & Lowe, 2006) (e.g., "Over the last 4 weeks, how often have you been feeling nervous, anxious or on edge?"), with response categories ranging from 0 (not at all) to 3 (nearly every day). The summary score was created by summing responses across all items, with higher scores representing more anxiety symptoms ($\alpha = 0.88$, range=0 to 21). The scale was validated in previous work, and demonstrated good psychometric properties (Spitzer et al., 2006).

Hopelessness. To assess hopelessness, we used an item from the CESD-10 scale (Andresen et al., 1994): "I felt hopeful about the future.", with response options ranging from 0 (rarely or none of the time) to 3 (all of the time). We then reverse scored the response, such that hopelessness was indicated by higher scores.

Loneliness. To assess loneliness we used an item from the CESD-10 scale (Andresen et al., 1994): “I felt lonely.”, with response options ranging from 0 (rarely or none of the time) to 3 (all the time). Responses were treated as a continuous score, with higher scores representing greater loneliness.

Heavy drinking. Heavy drinking was assessed by asking participants to respond to the following question: “In a typical month during the past year, what was the largest number of drinks of beer, wine, and/or liquor you may have had in one day.” Response options ranged from 1 (none) to 6 (15 or more). Heavy drinking was considered as those who reported having more than 5 drinks in a single day (Harrington et al., 2018; Naimi et al., 2003).

Cigarette smoking. Cigarette smoking was assessed through the following question: “Do you currently smoke cigarettes?”, with response options of “yes” or “no”.

Frequent physical activity. Frequent physical activity was assessed through validated questionnaires and a metabolic equivalent value (MET), following prior of Hu et al. (1999). MET scores ≥ 7.5 hours per week were considered the minimum recommended level of physical activity (US Department of Health Human Services, 2008).

Preventive healthcare use. Preventive healthcare use was assessed by a question asking participants if, in the past two years, they had a physical exam for screening purposes. Response options were “yes” or “no”.

Dietary quality. The Alternative-Healthy Eating Index (AHEI) was used to assess overall dietary quality (Chiuve et al., 2012) and was calculated based on data from the food frequency questionnaire (FFQ). Higher scores indicated better dietary quality.

All-cause mortality. All deaths that took place by the end of the 2015 questionnaire, regardless of cause, were evaluated via reports from next of kin, state vital records, or the national death index.

Number of physical health problems. Participants self-reported if they had received a diagnosis for any of the following conditions: Type 2 diabetes, stroke, coronary heart disease, and cancer. Medical records were used to validate self-reported diagnosis. In addition, BMI was calculated using self-reported height and weight, and then validated against objectively measured BMI in a subgroup of participants (r=0.97) (Rimm et al., 1990). BMI≥ 25 kg/m^2 was considered as overweight/obesity (WHO, 1995). A summary score of number of physical health

problems was created by summing the total number of following: diabetes, stroke, coronary heart disease, cancer, and overweight/obesity.

Table S1. Participant characteristics in the full sample (The Nurses' Health Study II 2001 to 2011, 2013 or 2015 Questionnaire Wave, N=54,703)

	Questionnaire wave	Mean (SD) or %
**Exposures** (2008 Supplementary Survey)		
Self-forgiveness	2008	
Never		2.18
Seldom		11.77
Often		44.38
Always/almost always		41.67
Divine forgiveness	2008	
Do not believe in god or a higher power		5.65
Never		1.30
Seldom		4.11
Often		18.84
Always/almost always		70.11
**Outcomes** (wave 2011, 2013 or 2015)		
Psychological well-being		
Positive affect (range: 0-3)	2013	2.16 (0.72)
Social integration (range: 0-12)	2013	6.44 (2.30)
Psychological distress (outcomes)		
Depressive symptoms (range: 0-30)	2013	5.84 (4.60)
Depression diagnosis	2015	9.87
Anxiety symptoms (range: 0-21)	2013	2.88 (3.42)
Hopelessness (range: 0-3)	2013	0.95 (0.96)
Loneliness (range: 0-3)	2013	0.47 (0.72)
Health behaviors		
Heavy drinking	2011	4.51
Current cigarette smoking	2015	4.04
Frequent physical activity	2013	75.48
Preventive healthcare use	2015	89.29
Dietary quality (range: 19.09 to 105.94)	2011	64.53 (13.03)
Physical health		
All-cause mortality	2015	1.37
No. of physical health problems (range: 0-5)	2015	0.83 (0.73)
Diabetes	2015	8.73
Stroke	2015	2.18
Heart Disease	2013	1.68
Cancer	2015	12.45
Overweight/obesity	2015	59.13
**Covariates** (wave 2008 or prior)		

Sociodemographic characteristics		
Age, in years (range: 43-64)	2008	53.37 (4.65)
Non-Hispanic White, %	2005	95.75
Married, %	2005	81.41
Geographic region, %	2007	
Northeast		31.94
Midwest		32.89
South		18.79
West		16.37
Subjective SES in the U.S. (range:1-10)	2001	7.11 (1.32)
Subjective SES in the community (range:1-10)	2001	6.93 (1.58)
Pretax household income	2001	
<$50,000		15.74
$50,000–$74,999		27.33
$75,000–$99,999		21.38
≥$100,000		35.54
Census tract median income, %	2001	
<$50,000		25.67
$50,000–$74,999		49.01
$75,000–$99,999		19.06
≥$100,000		6.27
Census tract college education rate (range:0-88%)	2001	0.32 (0.16)
Currently employed, %	2001	88.78
Childhood abuse victimization (range: 0-5)	2001	1.76 (1.50)
Rotating night shift work (over past 2 years), %	2005	
None		92.08
1-9 months		3.39
10-19 months		1.30
20+ months		3.23
Religious service attendance, %	2008	
Never		23.77
Less than once/week		35.76
At least once/week		40.48
Number of close friends (range: 0-5)	2008	1.73 (0.66)
Prior health status or prior health behaviors		
Prior positive affect (range: 0-3)	2008	2.10 (0.77)
Prior depressive symptoms (range: 0-30)	2008	6.05 (5.01)
Prior depression diagnosis, %	2007	15.15
Prior anxiety symptoms (range: 0-15)	2005	2.43 (2.22)
Prior hopelessness (range: 0-3)	2008	0.86 (0.92)
Prior alcohol intake, %	2007	
0 g/day		33.58

0.1-9.9 g/day		44.53
10.0-29.9 g/day		17.82
30+ g/day		4.07
Prior cigarette smoking, %	2007	
never smoker		65.86
former smoker		28.11
current smoker 1-14/d		3.34
current smoker 15-24/day		2.01
current smoker >=25/day		0.68
Prior body max index (kg/m^2), %	2007	
<20		5.47
20-24.9		38.10
25-29.9		29.53
30-34.9		15.14
35+		11.76
Prior physical activity (METS score), %	2005	
<3		15.98
3-8.9		18.94
9-17.9		20.65
18-26.9		14.06
>=27		30.37
Prior dietary quality (AHEI score), %	2007	
Bottom tertile		32.31
Middle tertile		33.33
Top tertile		34.36
Prior preventive healthcare use, %	2007	85.28
Diabetes, %	2008	4.62
CHD, %	2008	1.14
Stroke, %	2008	1.34
Cancer, %	2008	6.56
Postmenopausal status, %	2007	60.65
Replacement Hormone use, %	2007	14.42

Table S2. Participant characteristics (age-adjusted) by divine-forgiveness at study baseline (The Nurses' Health Study II 2008 Supplementary Survey, N=50,697)

	Divine forgiveness		
**Participant characteristics**	Never/ seldom (n=2,905)	Often (n=10,121)	Always/ almost always (n=37,671)
Age, in years (range: 43-64)[a]	53.65 (4.49)	53.19 (4.59)	53.28 (4.70)
Non-Hispanic white, %	95.59	96.62	95.48
Marital status, %	77.54	80.00	82.44
Geographic region, %			
- Northeast	39.76	38.75	29.43
- Midwest	23.85	30.20	35.53
- South	14.02	14.89	20.71
- West	22.37	16.16	14.33
Subjective SES in US (range: 1-10)	6.98 (1.45)	7.00 (1.28)	7.12 (1.30)
Subjective SES in community (range: 1-10)	6.61 (1.71)	6.73 (1.55)	6.99 (1.57)
Census-tract college education rate (range: 0-0.88)	0.36 (0.17)	0.33 (0.16)	0.30 (0.15)
Household income, %			
- <$50,000	15.20	14.57	16.44
- $50,000-$74,999	24.23	26.80	28.21
- $75,000-$99,999	20.36	21.43	21.79
- >=$100,000	40.21	37.20	33.56
Census tract median income, %			
- <$50,000	18.38	23.16	27.83
- $50,000-$74,999	46.72	48.95	49.52
- $75,000-$99,999	24.16	20.78	17.59
- >=$100,000	10.74	7.11	5.07
Night shift work over past 2 years, %			
- none	91.76	91.74	92.04
- 1-9 months	3.52	3.82	3.38
- 10-19 months	0.87	1.33	1.35
- 20+ months	3.85	3.11	3.23
Currently employed, %	89.21	90.31	88.40
Childhood abuse victimization (range: 0-5)	1.96 (1.57)	1.85 (1.50)	1.71 (1.49)
Religious service attendance, %			
- never/almost never	56.01	30.53	13.86
- <once/week	38.28	47.70	34.26
- >=once/week	5.71	21.77	51.87
Number of close friends (range: 0-5)	1.54 (0.72)	1.67 (0.66)	1.76 (0.65)
Depressive symptoms (range: 0-30)	8.50 (6.02)	7.10 (5.17)	5.55 (4.74)
Depression diagnosis, %	19.94	16.03	14.37
Anxiety symptoms (range: 0-15)	2.87 (2.55)	2.68 (2.29)	2.36 (2.18)
Hopelessness (range: 0-3)	1.25 (0.96)	1.04 (0.90)	0.78 (0.91)
Positive affect (range: 0-3)	1.76 (0.84)	1.94 (0.75)	2.17 (0.75)
Preventive healthcare use, %	82.28	84.60	85.83

Alcohol intake, %			
- 0 grams/day	26.03	27.02	36.93
- 0.1-9.9 grams/day	44.91	48.45	43.67
- 10.0-29.9 grams/day	22.76	20.19	16.04
- 30+ grams/day	6.29	4.35	3.36
Cigarette smoking, %			
- never smoker	57.77	61.15	68.71
- former smoker	33.49	31.79	25.73
- current smoker 1-14/day	4.73	3.85	3.09
- current smoker 15-24/day	2.92	2.39	1.87
- current smoker >=25/day	1.08	0.83	0.60
Physical activity (METS[b]), %			
- <3	16.91	15.13	16.44
- 3-8.9	18.17	18.72	19.43
- 9-17.9	19.29	20.91	20.72
- 18-26.9	13.03	13.97	14.06
- >=27	32.59	31.26	29.35
Dietary quality (AHEI score[c]), %			
- bottom tertile, %	31.17	32.62	33.35
- middle tetile, %	31.71	33.67	33.76
- top tertile, %	37.12	33.71	32.88
BMI categories (kg/m^2), %			
- <20	6.83	5.81	5.17
- 20-24.9	39.40	38.18	37.23
- 25-29.9	27.28	30.01	29.75
- 30-34.9	14.53	14.65	15.64
- 35+	11.96	11.35	12.21
Diabetes, %	3.87	4.38	4.88
CHD, %	1.18	1.08	1.18
Stroke, %	1.27	1.31	1.37
Cancer, %	6.45	6.59	6.54
Postmenopausal status, %	59.59	60.07	60.28
Replacement Hormone use, %	13.83	13.74	14.49

Notes: Values are means (SD) for continuous variables and percentages for categorical variables, and are standardized to the age distribution of the study population. Values of polytomous variables may not sum to 100% due to rounding.

[a] Value is not age adjusted.
[b] Metabolic equivalents score (METS) was used to measure physical activity.
[c] Alternate Healthy Eating Index (AHEI) was used to measure dietary quality.

Table S3. Sensitivity analyses on self-forgiveness and subsequent health and well-being in mid-life, restricting to participants free of major physical health problems at baseline (The Nurses' Health Study II 2008 supplementary survey to 2011, 2013 or 2015 questionnaire wave, N=47,757[a])

	**Self-forgiveness** [b]							
	Often vs. Never/seldom				Always/almost always vs. Never/seldom			
Health and well-being outcomes	RR[c]	β[d]	95% CI	P-value threshold	RR[c]	β[d]	95% CI	P-value threshold
**Psychosocial Well-being**								
Positive affect		0.12	0.09, 0.14	<.0026[e]		0.23	0.20, 0.26	<.0026[e]
Social integration		0.07	0.05, 0.09	<.0026[e]		0.12	0.09, 0.14	<.0026[e]
**Psychological Distress**								
Depression diagnosis	0.95		0.87, 1.03		0.91		0.83, 1.00	
Depressive symptoms		-0.13	-0.15, -0.10	<.0026[e]		-0.21	-0.23, -0.18	<.0026[e]
Anxiety symptoms		-0.02	-0.05, 0.01			-0.15	-0.18, -0.12	<.0026[e]
Hopelessness		-0.12	-0.15, -0.09	<.0026[e]		-0.18	-0.21, -0.15	<.0026[e]
Loneliness		-0.10	-0.13, -0.07	<.0026[e]		-0.12	-0.15, -0.09	<.0026[e]
**Health Behaviors**								
Heavy drinking	0.99		0.84, 1.16		0.97		0.81, 1.16	
Current cigarette smoking	0.95		0.79, 1.14		0.98		0.80, 1.20	
Frequent physical activity	1.00		0.97, 1.04		1.00		0.97, 1.04	
Preventive healthcare use	1.00		0.97, 1.03		1.00		0.96, 1.03	
Dietary quality		0.00	-0.03, 0.02			0.02	-0.01, 0.04	
**Physical Health**								
All-cause mortality	1.07		0.81, 1.43		1.15		0.85, 1.55	
No. of physical health problems		-0.01	-0.03, 0.01			0.00	-0.02, 0.03	
Diabetes	0.84		0.72, 0.97	<.05	0.92		0.79, 1.08	
Stroke	0.92		0.66, 1.28		0.90		0.63, 1.29	
Heart Disease	0.75		0.49, 1.16		1.30		0.84, 1.99	
Cancer	0.89		0.79, 1.00		0.92		0.81, 1.03	
Overweight/obesity	1.02		0.98, 1.05		1.02		0.98, 1.06	

Abbreviations: RR, risk ratio; CI, confidence interval.

[a] The full analytic sample was restricted to those who responded to the Nurses' Health Study II 2008 supplementary survey in which the exposure variable forgiveness was assessed. Multiple imputation was performed to impute missing data on all variables. Participants with major physical illness at baseline (n=6,946, including type 2 diabetes, stroke, heart diseases, and cancer) were excluded from the analyses.

[b] A set of generalized estimating equations were used to regress each outcome on forgiveness separately. All models controlled for participants' age, race, marital status, geographic region, childhood abuse, socioeconomic status (subjective SES, household income, census tract college education rate, and census tract median income), employment status, night shift work schedule, religious service attendance, number of close friends, prior health status or health behaviors (prior depressive symptoms, depression diagnosis, anxiety symptoms, hopelessness, positive affect, dietary quality, body mass index, smoking, alcohol intake, physical activity, preventive healthcare use, postmenopausal status, and menopausal hormone therapy use).

[c] The effect estimates for the outcomes of heavy drinking, current smoking, mortality, diabetes, heart diseases, stroke and cancer were odds ratio. These outcomes were rare [prevalence<10%], so the odds ratio would approximate RR. Effect estimates for other dichotomized outcomes were RR.

[d] All continuous outcomes were standardized (mean=0, standard deviation=1), and β was the standardized effect size.

[e] p<0.05 after Bonferroni correction (the p value cutoff for Bonferroni correction is p=0.05/19 outcomes=0.0026)

Table S4. Complete-case analysis on self-forgiveness and subsequent health and well-being (The Nurses' Health Study II 2008 to 2011, 2013 or 2015 questionnaire wave, N=36,749 to 41,747[a])

	Self-forgiveness [b]							
	Often vs. Never/seldom				Always/almost always vs. Never/seldom			
Health and well-being outcomes	RR[c]	β[d]	95% CI	P-value threshold	RR[c]	β[d]	95% CI	P-value threshold
**Psychosocial Well-being**								
Positive affect		0.11	0.09, 0.14	<.0026[e]		0.23	0.20, 0.26	<.0026[e]
Social integration		0.06	0.04, 0.09	<.0026[e]		0.11	0.08, 0.13	<.0026[e]
**Psychological Distress**								
Depression diagnosis	0.96		0.88, 1.05		0.92		0.83, 1.01	
Depressive symptoms		-0.13	-0.15, -0.10	<.0026[e]		-0.21	-0.24, -0.18	<.0026[e]
Anxiety symptoms		-0.16	-0.18, -0.13	<.0026[e]		-0.23	-0.26, -0.20	<.0026[e]
Hopelessness		-0.11	-0.15, -0.08	<.0026[e]		-0.18	-0.21, -0.15	<.0026[e]
Loneliness		-0.10	-0.12, -0.07	<.0026[e]		-0.12	-0.16, -0.09	<.0026[e]
**Health Behaviors**								
Heavy drinking	0.98		0.82, 1.18		0.93		0.76, 1.12	
Current cigarette smoking	1.05		0.86, 1.29		1.14		0.92, 1.42	
Frequent physical activity	1.01		0.97, 1.04		1.01		0.97, 1.05	
Preventive healthcare use	1.00		0.97, 1.03		1.00		0.96, 1.03	
Dietary quality		-0.01	-0.03, 0.02			0.02	-0.01, 0.04	
**Physical Health**								
All-cause mortality	1.21		0.92, 1.58		1.46		1.09, 1.95	
No. of physical health problems		0.00	-0.02, 0.02			0.00	-0.02, 0.02	
Diabetes	0.84		0.72, 0.99	<.05	1.01		0.85, 1.19	
Stroke	1.00		0.71, 1.42		0.91		0.62, 1.32	
Heart Disease	0.83		0.53, 1.29		1.33		0.85, 2.09	
Cancer	0.96		0.88, 1.05		0.96		0.88, 1.06	
Overweight/obesity	1.02		0.98, 1.06		1.02		0.97, 1.06	

Abbreviations: RR, risk ratio; CI, confidence interval.

[a] The full analytic sample was restricted to those who responded to the Nurses' Health Study II 2008 supplementary survey in which the exposure variable forgiveness was assessed and had data on forgiveness, all covariates and the outcome variable under investigation.

[b] A set of regression models were used to regress each outcome on forgiveness separately. All models controlled for participants' age, race, marital status, geographic region, childhood abuse, socioeconomic status (subjective SES, census tract college education rate, and census tract median income), employment status, night shift work schedule, religious service attendance, number of close friends, prior health status or health behaviors (prior depressive symptoms, depression diagnosis, anxiety symptoms, hopelessness, positive affect, dietary quality, body mass index, smoking, alcohol intake, physical activity, preventive healthcare use, postmenopausal status, menopausal hormone therapy use, history of diabetes, heart diseases, stroke, and cancer).

[c] The effect estimates for the outcomes of heavy drinking, current smoking, mortality, diabetes, heart diseases, stroke and cancer were odds ratio. These outcomes were rare [prevalence<10%], so the odds ratio would approximate RR. Effect estimates for other dichotomized outcomes were RR.

[d] All continuous outcomes were standardized (mean=0, standard deviation=1), and β was the standardized effect size.

[e] $p<0.05$ after Bonferroni correction (the p value cutoff for Bonferroni correction is $p=0.05/19$ outcomes=0.0026)

Table S5. Complete-case analysis on self-forgiveness and divine-forgiveness and incidence of physical health problems (The Nurses' Health Study II 2008 to 2015 questionnaire wave [a])

		Self-forgiveness [b]					
		Often vs. Never/seldom			Always/almost always vs. Never/seldom		
Incident health outcomes	N	RR[c]	95% CI	P-value	RR[c]	95% CI	P-value
Diabetes	38,283	0.84	0.72, 0.99	0.038	1.01	0.85, 1.19	0.939
Stroke	39,480	1.00	0.71, 1.42	0.999	0.91	0.62, 1.32	0.605
Heart Disease	39,955	0.83	0.53, 1.29	0.403	1.33	0.85, 2.09	0.215
Cancer	37,600	0.91	0.80, 1.04	0.155	0.92	0.80, 1.06	0.235
Overweight/obesity	17,360	1.10	0.98, 1.23	0.100	1.07	0.95, 1.21	0.251

		Divine-forgiveness [b]					
		Often vs. Never/seldom			Always/almost always vs. Never/seldom		
Incident health outcomes	N	RR[c]	95% CI	P-value	RR[c]	95% CI	P-value
Diabetes	36,373	0.86	0.67, 1.09	0.216	0.82,	0.65, 1.13	0.089
Stroke	37,527	0.82	0.49, 1.39	0.465	0.84	0.51, 1.38	0.487
Heart Disease	37,988	0.73	0.40, 1.35	0.320	0.67	0.37, 1.19	0.170
Cancer	35,751	1.03	0.84, 1.27	0.778	1.01	0.83, 1.24	0.884
Overweight/obesity	16,317	1.31	1.09, 1.58	0.005	1.40	1.17, 1.68	<.001

Abbreviations: RR, risk ratio; CI, confidence interval.

[a] The full analytic sample was restricted to those who responded to the Nurses' Health Study II 2008 supplementary survey in which the exposure variable forgiveness was assessed and had data on forgiveness, all covariates and the outcome variable under investigation. Participants with the corresponding physical health problem at baseline were removed from each analysis, so that the analyses were examining forgiveness in relation to incidence of each physical health problem.

[b] A set of regression models were used to regress each outcome on forgiveness separately. All models controlled for participants' age, race, marital status, geographic region, childhood abuse, socioeconomic status (subjective SES, census tract college education rate, and census tract median income), employment status, night shift work schedule, religious service attendance, number of close friends, prior health status or health behaviors (prior depressive symptoms, depression diagnosis, anxiety symptoms, hopelessness, positive affect, dietary quality, smoking, alcohol intake, physical activity, preventive healthcare use, postmenopausal status, and menopausal hormone therapy use). The following baseline characteristics were also either adjusted for as covariates or participants with these conditions at baseline were removed from the analyses in each model, depending on the outcome under investigation: body mass index, history of diabetes, history of heart diseases, history of stroke, and history of cancer. w

[c] The effect estimates for the outcomes of diabetes, heart diseases and stroke were odds ratio. These outcomes were rare [prevalence<10%], so the odds ratio would approximate RR. Effect estimates for cancer and overweight/obesity were RR.

Supplementary Table S6. Self-forgiveness (standardized score) and subsequent health and well-being in mid-life (The Nurses' Health Study II 2008 supplementary survey to 2011, 2013 or 2015 questionnaire wave, N=54,703[a])

	**Self-forgiveness** (standardized score)			
Health and well-being outcomes	RR[c]	β[d]	95% CI	P-value threshold
**Psychosocial Well-being**				
Positive affect		0.08	0.07, 0.09	<.0026[e]
Social integration		0.04	0.03, 0.04	<.0026[e]
**Psychological Distress**				
Depression diagnosis	0.98		0.95, 1.00	
Depressive symptoms		-0.07	-0.08, -0.06	<.0026[e]
Anxiety symptoms		-0.06	-0.07, -0.05	<.0026[e]
Hopelessness		-0.06	-0.07, -0.05	<.0026[e]
Loneliness		-0.04	-0.05, -0.03	<.0026[e]
**Health Behaviors**				
Heavy drinking	0.99		0.94, 1.05	
Current cigarette smoking	0.98		0.92, 1.04	
Frequent physical activity	1.00		0.99, 1.01	
Preventive healthcare use	1.00		0.99, 1.01	
Dietary quality		0.01	0.00, 0.02	<.05
**Physical Health**				
All-cause mortality	1.11		1.02, 1.21	<.05
No. of physical health problems		0.00	-0.00, 0.01	
Diabetes	1.02		0.97, 1.07	
Stroke	1.00		0.89, 1.11	
Heart Disease	1.17		1.02, 1.35	<.05
Cancer	0.99		0.97, 1.02	
Overweight/obesity	1.00		0.99, 1.02	

Abbreviations: RR, risk ratio; CI, confidence interval.

[a] The full analytic sample was restricted to those who responded to the Nurses' Health Study II 2008 supplementary survey in which the exposure variable forgiveness was assessed. Multiple imputation was performed to impute missing data on all variables.

[b] A set of generalized estimating equations were used to regress each outcome on forgiveness (standardized score, mean=0, standard deviation=1) separately. All models controlled for participants' age, race, marital status, geographic region, childhood abuse, socioeconomic status (subjective SES, household income, census tract college education rate, and census tract median income), employment status, night shift work schedule, religious service attendance, number of close friends, prior health status or health behaviors (prior depressive symptoms, depression diagnosis, anxiety symptoms, hopelessness, positive affect, dietary quality, body mass index, smoking, alcohol intake, physical activity, preventive healthcare use, postmenopausal status, menopausal hormone therapy use, history of diabetes, heart diseases, stroke, and cancer).

[c] The effect estimates for the outcomes of heavy drinking, current smoking, mortality, diabetes, heart diseases, stroke and cancer were odds ratio. These outcomes were rare [prevalence<10%], so the odds ratio would approximate RR. Effect estimates for other dichotomized outcomes were RR.

[d] All continuous outcomes were standardized (mean=0, standard deviation=1), and β was the standardized effect size.

[e] $p<0.05$ after Bonferroni correction (the p value cutoff for Bonferroni correction is $p=0.05/19$ outcomes=0.0026)

Table S7. Complete-case analysis on divine-forgiveness and subsequent health and well-being (The Nurses' Health Study II 2008 to 2011, 2013 or 2015 questionnaire wave, N=34,916 to 39,712[a])

	Divine forgiveness [b]							
	Often vs. Never/seldom				Always/almost always vs. Never/seldom			
Health and well-being outcomes	RR[c]	β[d]	95% CI	P-value threshold	RR[c]	β[d]	95% CI	P-value threshold
**Psychosocial Well-being**								
Positive affect		0.08	0.04, 0.13	<.0026[e]		0.20	0.15, 0.24	<.0026[e]
Social integration		0.01	-0.02, 0.05			0.10	0.07, 0.14	<.0026[e]
**Psychological Distress**								
Depression diagnosis	1.01		0.88, 1.16		1.03		0.90, 1.18	
Depressive symptoms		-0.07	-0.11, -0.03	<.0026[e]		-0.15	-0.19, -0.11	<.0026[e]
Anxiety symptoms		-0.06	-0.10, -0.02	<.01		-0.12	-0.16, -0.08	<.0026[e]
Hopelessness		-0.07	-0.11, -0.02	<.01		-0.16	-0.21, -0.12	<.0026[e]
Loneliness		-0.06	-0.10, -0.01	<.05		-0.11	-0.16, -0.07	<.0026[e]
**Health Behaviors**								
Heavy drinking	0.92		0.72, 1.19		1.05		0.83, 1.34	
Current cigarette smoking	1.34		0.98, 1.82		1.27		0.95, 1.70	
Frequent physical activity	0.98		0.93, 1.04		0.97		0.92, 1.02	
Preventive healthcare use	1.01		0.96, 1.06		1.01		0.96, 1.06	
Dietary quality		-0.03	-0.07, 0.00			-0.03	-0.07, 0.00	
**Physical Health**								
All-cause mortality	0.91		0.60, 1.37		1.16		0.79, 1.70	
No. of physical health problems		0.02	-0.01, 0.05			0.03	0.00, 0.06	<.05
Diabetes	0.86		0.67, 1.09		0.82		0.65, 1.03	
Stroke	0.82		0.49, 1.39		0.84		0.51, 1.38	
Heart Disease	0.73		0.40, 1.35		0.67		0.37, 1.19	
Cancer	1.01		0.88, 1.16		1.01		0.88, 1.15	
Overweight/obesity	1.05		0.98, 1.12		1.06		1.00, 1.13	

Abbreviations: RR, risk ratio; CI, confidence interval.

[a] The full analytic sample was restricted to those who responded to the Nurses' Health Study II 2008 supplementary survey in which the exposure variable forgiveness was assessed and had data on forgiveness, all covariates and the outcome variable under investigation. Participants who reported not believe in God or a higher power were removed from the analyses.

[b] A set of regression models were used to regress each outcome on forgiveness separately. All models controlled for participants' age, race, marital status, geographic region, childhood abuse, socioeconomic status (subjective SES, census tract college education rate, and census tract median income), employment status, night shift work schedule, religious service attendance, number of close friends, prior health status or health behaviors (prior depressive symptoms, depression diagnosis, anxiety symptoms, hopelessness, positive affect, dietary quality, body mass index, smoking, alcohol intake, physical activity, preventive healthcare use, postmenopausal status, menopausal hormone therapy use, history of diabetes, heart diseases, stroke, and cancer).

[c] The effect estimates for the outcomes of heavy drinking, current smoking, mortality, diabetes, heart diseases, stroke and cancer were odds ratio. These outcomes were rare [prevalence<10%], so the odds ratio would approximate RR. Effect estimates for other dichotomized outcomes were RR.

[d] All continuous outcomes were standardized (mean=0, standard deviation=1), and β was the standardized effect size.

[e] p<0.05 after Bonferroni correction (the p value cutoff for Bonferroni correction is p=0.05/19 outcomes=0.0026)

Table S8. Sensitivity analyses on divine-forgiveness and subsequent health and well-being in mid-life, restricting to participants free of major physical health problems at baseline (The Nurses' Health Study II 2008 supplementary survey to 2011, 2013 or 2015 questionnaire wave, N=45,054[a])

	Divine forgiveness[b]							
	Often vs. Never/seldom				Always/almost always vs. Never/seldom			
Health and well-being outcomes	RR[c]	β[d]	95% CI	P-value threshold	RR[c]	β[d]	95% CI	P-value threshold
**Psychosocial Well-being**								
Positive affect		0.07	0.03, 0.12	<.0026[e]		0.18	0.14, 0.22	<.0026[e]
Social integration		0.03	-0.01, 0.06			0.12	0.09, 0.15	<.0026[e]
**Psychological Distress**								
Depression diagnosis	1.01		0.89, 1.15		1.05		0.93, 1.19	
Depressive symptoms		-0.09	-0.13, -0.04	<.0026[e]		-0.15	-0.19, -0.12	<.0026[e]
Anxiety symptoms		0.04	-0.01, 0.09			-0.03	-0.07, 0.01	
Hopelessness		-0.08	-0.12, -0.03	<.0026[e]		-0.16	-0.20, -0.12	<.0026[e]
Loneliness		-0.07	-0.11, -0.02	<.01		-0.12	-0.16, -0.07	<.0026[e]
**Health Behaviors**								
Heavy drinking	0.94		0.75, 1.18		1.03		0.83, 1.27	
Current cigarette smoking	1.25		0.94, 1.67		1.13		0.86, 1.48	
Frequent physical activity	1.00		0.95, 1.05		0.98		0.93, 1.03	
Preventive healthcare use	1.01		0.97, 1.06		1.01		0.97, 1.06	
Dietary quality		-0.03	-0.06, 0.01			-0.03	-0.06, 0.01	
**Physical Health**								
All-cause mortality	0.87		0.57, 1.32		1.02		0.69, 1.51	
No. of physical health problems		0.02	-0.01, 0.06			0.04	0.01, 0.08	<.05
Diabetes	0.85		0.67, 1.06		0.87		0.70, 1.08	
Stroke	0.80		0.47, 1.37		1.15		0.70, 1.89	
Heart Disease	0.59		0.32, 1.08		0.69		0.39, 1.21	
Cancer	0.94		0.78, 1.12		0.96		0.80, 1.14	
Overweight/obesity	1.05		0.99, 1.12		1.07		1.01, 1.13	<.05

Abbreviations: RR, risk ratio; CI, confidence interval.

[a] The full analytic sample was restricted to those who responded to the Nurses' Health Study II 2008 supplementary survey in which the exposure variable forgiveness was assessed. Participants who reported not believe in God or a higher power were removed from the analyses. Multiple imputation was performed to impute missing data on all variables. Participants with major physical illness at baseline (n=6,607, including type 2 diabetes, stroke, heart diseases, and cancer) were excluded from the analyses.

[b] A set of generalized estimating equations were used to regress each outcome on forgiveness separately. All models controlled for participants' age, race, marital status, geographic region, childhood abuse, socioeconomic status (subjective SES, household income, census tract college education rate, and census tract median income), employment status, night shift work schedule, religious service attendance, number of close friends, prior health status or health behaviors (prior depressive symptoms, depression diagnosis, anxiety symptoms, hopelessness, positive affect, dietary quality, body mass index, smoking, alcohol intake, physical activity, preventive healthcare use, postmenopausal status, and menopausal hormone therapy use).

[c] The effect estimates for the outcomes of heavy drinking, current smoking, mortality, diabetes, heart diseases, stroke and cancer were odds ratio. These outcomes were rare [prevalence<10%], so the odds ratio would approximate RR. Effect estimates for other dichotomized outcomes were RR.

[d] All continuous outcomes were standardized (mean=0, standard deviation=1), and β was the standardized effect size.

[e] p<0.05 after Bonferroni correction (the p value cutoff for Bonferroni correction is p=0.05/19 outcomes=0.002

Supplementary Table S9. Divine-forgiveness (standardized score) and subsequent health and well-being in mid-life (The Nurses' Health Study II 2008 supplementary survey to 2011, 2013 or 2015 questionnaire wave, N=51,661[a])

	**Self-forgiveness** (standardized score)			
Health and well-being outcomes	RR[c]	β[d]	95% CI	P-value threshold
**Psychosocial Well-being**				
Positive affect		0.06	0.05, 0.06	<.0026[e]
Social integration		0.04	0.03, 0.05	<.0026[e]
**Psychological Distress**				
Depression diagnosis	1.02		0.99, 1.05	
Depressive symptoms		-0.04	-0.05, -0.03	<.0026[e]
Anxiety symptoms		-0.02	-0.03, -0.01	<.0026[e]
Hopelessness		-0.05	-0.06, -0.04	<.0026[e]
Loneliness		-0.03	-0.04, -0.02	<.0026[e]
**Health Behaviors**				
Heavy drinking	1.04		0.98, 1.09	
Current cigarette smoking	0.99		0.93, 1.05	
Frequent physical activity	0.99		0.98, 1.00	
Preventive healthcare use	1.00		0.99, 1.01	
Dietary quality		-0.01	-0.01, 0.00	
**Physical Health**				
All-cause mortality	1.05		0.97, 1.15	
No. of physical health problems		0.01	0.00, 0.01	<.05
Diabetes	0.98		0.93, 1.03	
Stroke	1.07		0.95, 1.20	
Heart Disease	0.96		0.84, 1.09	
Cancer	1.00		0.97, 1.02	
Overweight/obesity	1.01		1.00, 1.03	

Abbreviations: RR, risk ratio; CI, confidence interval.

[a] The full analytic sample was restricted to those who responded to the Nurses' Health Study II 2008 supplementary survey in which the exposure variable forgiveness was assessed. Participants who reported not believe in God or a higher power were removed from the analyses. Multiple imputation was performed to impute missing data on all variables.

[b] A set of generalized estimating equations were used to regress each outcome on forgiveness (standardized score, mean=0, standard deviation=1) separately. All models controlled for participants' age, race, marital status, geographic region, childhood abuse, socioeconomic status (subjective SES, household income, census tract college education rate, and census tract median income), employment status, night shift work schedule, religious service attendance, number of close friends, prior health status or health behaviors (prior depressive symptoms, depression diagnosis, anxiety symptoms, hopelessness, positive affect, dietary quality, body mass index, smoking, alcohol intake, physical activity, preventive healthcare use, postmenopausal status, menopausal hormone therapy use, history of diabetes, heart diseases, stroke, and cancer).

[c] The effect estimates for the outcomes of heavy drinking, current smoking, mortality, diabetes, heart diseases, stroke and cancer were odds ratio. These outcomes were rare [prevalence<10%], so the odds ratio would approximate RR. Effect estimates for other dichotomized outcomes were RR.

[d] All continuous outcomes were standardized (mean=0, standard deviation=1), and β was the standardized effect size.

[e] p<0.05 after Bonferroni correction (the p value cutoff for Bonferroni correction is p=0.05/19 outcomes=0.0026)

REFERENCES FOR THE SUPPLEMENTARY TEXT

Andresen, E. M., Malmgren, J. A., Carter, W. B., & Patrick, D. L. (1994). Screening for depression in well older adults: evaluation of a short form of the CES-D (Center for Epidemiologic Studies Depression Scale). *Am J Prev Med, 10*(2), 77-84.

Berkman, L. F., & Syme, S. L. (1979). Social networks, host resistance, and mortality: a nine-year follow-up study of Alameda County residents. *Am J Epidemiol, 109*(2), 186-204.

Chang, S. C., Glymour, M., Cornelis, M., Walter, S., Rimm, E. B., Tchetgen Tchetgen, E., . . . Kubzansky, L. D. (2017). Social Integration and Reduced Risk of Coronary Heart Disease in Women: The Role of Lifestyle Behaviors. *Circ Res, 120*(12), 1927-1937. doi:10.1161/CIRCRESAHA.116.309443

Chiuve, S. E., Fung, T. T., Rimm, E. B., Hu, F. B., McCullough, M. L., Wang, M., . . . Willett, W. C. (2012). Alternative dietary indices both strongly predict risk of chronic disease. *J Nutr, 142*(6), 1009-1018. doi:10.3945/jn.111.157222

Harrington, L. B., Hagan, K. A., Mukamal, K. J., Kang, J. H., Kim, J., Crous-Bou, M., . . . Jensen, M. K. (2018). Alcohol consumption and the risk of incident pulmonary embolism in US women and men. *J Thromb Haemost, 16*(9), 1753-1762. doi:10.1111/jth.14224

Hu, F. B., Sigal, R. J., Rich-Edwards, J. W., Colditz, G. A., Solomon, C. G., Willett, W. C., . . . Manson, J. E. (1999). Walking compared with vigorous physical activity and risk of type 2 diabetes in women: a prospective study. *JAMA, 282*(15), 1433-1439.

Naimi, T. S., Brewer, R. D., Mokdad, A., Denny, C., Serdula, M. K., & Marks, J. S. (2003). Binge Drinking Among US Adults. *JAMA, 289*(1), 70-75. doi:10.1001/jama.289.1.70

Rimm, E. B., Stampfer, M. J., Colditz, G. A., Chute, C. G., Litin, L. B., & Willett, W. C. (1990). Validity of self-reported waist and hip circumferences in men and women. *Epidemiology, 1*(6), 466-473.

Spitzer, R. L., Kroenke, K., Williams, J. B., & Lowe, B. (2006). A brief measure for assessing generalized anxiety disorder: the GAD-7. *Arch Intern Med, 166*(10), 1092-1097. doi:10.1001/archinte.166.10.1092

US Department of Health Human Services. (2008). *2008 physical activity guidelines for Americans*. Retrieved from Washington (District of Columbia): https://health.gov/paguidelines/2008/pdf/paguide.pdf

WHO. (1995). *Physical status: the use and interpretation of anthropometry. Report of a WHO Expert Committee* (0512-3054 (Print)). Retrieved from http://www.ncbi.nlm.nih.gov/pubmed/8594834
